# CMR quantification of infarct tissue heterogeneity and remote myocardial fibrotic burden during convalescent phase following acute myocardial infarction (MI) provided strong and complementary evidence of ventricular arrhythmogenicity from quantitative microvolt T-wave alternans testing (the NHLBI PROSPECT-CMR study)

**DOI:** 10.1186/1532-429X-14-S1-O18

**Published:** 2012-02-01

**Authors:** Bobby Heydari, Shuaib Abdullah, Evan Appelbaum, Damien Mandry, Ron Blankstein, Yucheng Chen, Jiazuo H Feng, Karl-Philipp Kienle, Elliott Antman, Heidi S Lumish, Sanjeev Francis, Henry Gewirtz, Udo Hoffmann, Daniel Forman, Lahn Fendelander, Roger Plaisted, Rob J van der Geest, Michael Jerosch-Herold, Raymond Kwong

**Affiliations:** 1 Brigham and Women's Hospital, Boston, MA, USA; 2 Internal Medicine, North Texas VA Medical Center, Bonham, TX, USA; 3 Cardiology, Massachusetts General Hospital, Boston, MA, USA; 4 Cambridge Heart, Cambridge, MA, USA; 5 Leiden University Medical Center, Leiden, Netherlands

## Summary

In patients with recent MI, elevated fibrotic burden within the remote myocardium quantified by CMR is strongly associated with post-exercise heart rate variability (HRV) and QT dispersion (QTD). In contrast, infarct tissue heterogeneity demonstrated strong association with prolonged mean QRS duration at rest and during exercise. We postulate that these patterns of ischemic structural/arrhythmogenic affiliations during convalescent infarct healing, likely reflect differences in depolarization/repolarization characteristics of different post-ischemic myocardium and sympathetic innervation.

## Background

Risk of sudden cardiac death (SCD) is highest during the first 6 months following acute MI. Infarct tissue heterogeneity characterized by CMR has been shown to be associated with arrhythmogenic substrates and SCD. In the remote myocardium, it is unclear whether development of increased fibrotic content as a result of ventricular remodeling is of arrhythmogenic significance. We tested the hypothesis that myocardial status after an ischemic event had specific patterns of arrhythmogenicity.

## Methods

We studied 85 patients (65 males) with CMR serially at 2-4 weeks and 6-months post-MI. During each visit we performed microvolt T-wave alternans (MTWA) testing using high resolution, ultra-sensitive electrodes. We assessed for arrhythmogenic markers including MTWA presence, QT dispersion (QTD), JT dispersion (JTD), mean QRS duration, and heart-rate variability (HRV). Post-processing of these markers was stratified to stages of exercise (rest, exercise, and post-exercise recovery). Total LGE size defined infarct size (in grams) and was subdivided into infarct core (CORE) and peri-infarct zone (PIZ) by previously described method. Fibrotic burden of remote myocardium was quantified with serial R1 mapping between myocardium and blood pool, up to 30 minutes after contrast administration. Fibrotic burden (normalized to hematocrit) was expressed as mean segmental fibrotic index (FIMeanSeg) and total fibrotic burden (FITotal).

## Results

The association of myocardial fibrotic burden and infarct tissue characteristics with arrhythmogenic markers are shown in Table 1.

**Table 1:** Association of CMR characteristics with markers of arrhythmogenicity

Association of fibrosis within remote myocardium with abnormal repolarization		
**Baseline**	**HRV (post-exercise)**	
	
FIMeanSeg	r=0.49 (p=0.03)	
FITotal	r=0.68 (p<0.001)	
	
**6-months**	**QT Dispersion (post-exercise)**	
	
FIMeanSeg	r=0.72 (p=0.0007)	

**Association of infarct tissue characteristics with abnormal depolarization**		

**Baseline**	**QRS duration (at rest)**	**QRS duration (during exercise)**

Total Infarct (g)	r=0.44 (p<0.001)	r=0.35 (p=0.006)
CORE (g)	r=0.45 (p=0.0001)	r=0.41 (p=0.0002)
PIZ (g)	r=0.37 (p=0.0008)	r=0.35 (p=0.0002)

Fibrotic burden at baseline and 6 months following MI were strongly correlated with HRV (post-exercise) and QTD (post-exercise), respectively. Infarct tissue heterogeneity at baseline was strongly associated with prolongation of mean QRS duration during both rest and exercise.

## Conclusions

Myocardial fibrotic burden was strongly associated with markers of abnormal repolarization. On the other hand, infarct tissue characteristics demonstrated strong correlation with abnormal electromyocardial depolarization. These findings support the notion that characterizing infarct tissue heterogeneity and non-infarcted myocardial fibrotic content may provide complementary information in advancing the knowledge of SCD risk in ischemic heart disease.

## Funding

National Heart Lung and Blood Institute, National Institutes of Health (RO1-HL091157).

Dr. Heydari's salary is supported by the Alberta Heritage Foundation for Medical Research.

